# Socioeconomic inequalities in the uptake of maternal healthcare services in Ethiopia

**DOI:** 10.1186/s12913-017-2298-9

**Published:** 2017-05-22

**Authors:** Markos Mezmur, Kannan Navaneetham, Gobopamang Letamo, Hadgu Bariagaber

**Affiliations:** Department of Population Studies, University of Botswana, Private Bag: UB 705, Gaborone, Botswana

**Keywords:** Socioeconomic Inequalities, Maternal Health Services, EDHS, Ethiopia

## Abstract

**Background:**

The progress in coverage of maternal health services in Ethiopia has been rather slow over the past decade and consequently the maternal mortality ratio was very high (673 per 100,000 live births) among the countries in Sub-Saharan Africa and remained constant during 2005–11 period. Earlier studies have mostly focused on determinants of maternal health seeking behavior in Ethiopia. However, little is known about the inequality aspects. This study intends to examine socioeconomic inequalities in the uptake of maternal health services and to identify factors that contribute to such inequalities.

**Methods:**

Data for the study is drawn from three rounds (year 2000, 2005 and 2011) of the Ethiopian Demographic and Health Surveys (EDHS). Concentration curves and the related concentration index (CI) were used to capture inequalities across the full range of socioeconomic status and highlight trends in the uptake of maternal health services in the country. Decomposition analysis was also employed to identify dominant factors that contribute to inequalities in the uptake of maternal healthcare services.

**Results:**

In this study, there is a general improvement in the uptake of maternal health services in Ethiopia over the past decade which is inequitable to the disadvantage of the poor. Inequalities are much larger in care during giving birth than in other maternal healthcare indicators. Furthermore, despite the progress made in reducing inequalities in the uptake of four antenatal care consultation (ANC) and tetanus toxoid (TT) injection, inequalities in access to health facilities for delivery and skilled assistance during delivery have rather widened over the same period. In all the survey years, inequalities in education and media access significantly contribute to inequalities in maternal health service utilization favoring the non-poor.

**Conclusion:**

The challenges to improving the uptake of maternal healthcare services in Ethiopia go beyond improving coverage of the maternal health services. Thus, addressing socioeconomic inequalities in accessing maternal health services is central to resolving challenges of maternal health. Furthermore, as Ethiopia moves forward with the sustainable development agenda, socioeconomic inequalities in uptake of maternal health services should also be continuously monitored.

## Background

Universal access to health services is seldom approached in most developing countries. Thus, healthcare services are more accessible to the better-off while healthcare benefits for the poor remain regressive [1, 2]. Despite the poor-rich gap that is larger for higher levels of care, inequalities in maternal health services remain a rule rather than an exception [3, 4]. For example, studies in developing countries have documented that maternal health service utilization is higher for women from the richest and richer households compared to those from the poorest households [5–12].

Inequalities by other socio-geographic measures such as education and urban-rural residence also account differentials in the uptake of such services. For example, studies have shown the positive association between education inequality and the uptake of antenatal care (ANC) [7, 13–17]; delivery in health facilities [14, 16–19]; and skilled delivery assistance [7, 11, 14]. Differentials in the diffusion of modern medical services and attitude towards modern medicine also vary across urban-rural dichotomy with urban areas having enhanced access and better-equipped services as living in rural areas in developing countries may mean residing in deprived communities in terms of social amenities and infrastructure [12–14, 19–22].

With an estimated population of slightly over 83 million in the year 2010, Ethiopia is the second most populous country in the African region [23]. The country has made a remarkable progress in reducing poverty over the past decade. The proportion of population below the national poverty line declined from 44% in 2000 to below 30% in 2011. Poverty incidence by a measure of international extreme poverty line (US$1.25 PPP) also fell from 55 to 31% [24].

Despite the progress in reducing poverty over the past decade, the country’s per capita national health expenditure of 20.77 US$ in the year 2011 remain the lowest compared to the Sub-Saharan Africa average of US$ 93.65 and that of WHO’s recommended US$ 30–40 per person needed to cover essential healthcare. In contrast, out-of pocket healthcare payment is catastrophic and comprises 80% of the healthcare expenditure. The figure is much higher compared to the 62% in Sub-Saharan Africa [25].

Maternal mortality in the country is one of the highest in Sub-Saharan Africa [26]. Though the maternal mortality ratio declined from 871 per 100,000 live births in 2000 to 673 in 2005, it remained stagnant thereafter [27]. The maternal mortality ratio in the year 2011 constituted 676 per 100,000 live births suggesting no improvement. Studies have examined maternal health seeking behavior in Ethiopia [28–30]. However, these studies focus on inter-individual variability with respect to individual level outcome with little systematic assessment of inequalities by socioeconomic group differences. The downside is that such studies did not make attempt to quantify socioeconomic inequalities in the comprehensive uptake of maternal health service indicators.

This study contribute to the current literature on socioeconomic related inequalities in the uptake of maternal health services in Ethiopia using a comprehensive set of maternal healthcare indicators across multiple surveys using concentration curve, concentration index and applied decomposition analysis to identify the contribution of inequality in endowment factors on the uptake of maternal health services in the country.

## Methods

### Data

Data for the study are drawn from three rounds (year 2000, 2005 and 2011) of EDHS. The surveys design used two-stage stratified-cluster sampling based primary sampling units (PSUs) of the 1994 and the 2007 Population and Housing Censuses of the country. PSUs were selected with probability proportional to size. For regions with very small population, the probability proportional to size allocated a very small sample size and PSUs in these regions were selected with equal size allocation. Given the predominantly rural population of the country and in order for the survey precision in urban areas be comparable with rural areas urban areas were slightly over sampled.

Stratification in the first stage was achieved separating each region into urban and rural areas. Sampling frame for selection of households in the second stage was derived from household listing operation carried out in all the selected PSUs. The result was a nationally representative sample of 14, 642 households and 15, 716 women in the year 2000, 14, 645 households and 14, 717 women in the year 2005, 18, 720 households and 17, 385 eligible women in the year 2011. The final sample size for this study constituted of 7, 917 women in the year 2000; 7, 273 women in the year 2005 and 7, 836 women in the year 2011 who had a live birth in the 5 years preceding the surveys. Details of the sampling procedure are present in survey reports available from Measure Demographic Health Survey (DHS) website (https://www.dhsprogram.com).

### Outcome and exposure variables

Outcome variables for the study are binary response data indicating whether a woman had received a minimum of one ANC, four or more ANC, initiated the first ANC in the first trimester of pregnancy, received two or more doses of tetanus toxoid (TT) injection and delivered in health facilities and was assisted by skilled health personnel during delivery for the most recent live birth in the 5 years preceding the surveys. Individual level exposure variables and their constructions are given in Table 1 the selection of which is justified by their empirical significance from existing literature [31–33].

**Table 1:** Description of exposure variables

Variable description	
Age Group	Re-coded into four categories of 15–19 coded as 1, 20–29 coded as 2, 30–39 coded as 3 and 40–49 coded as 4.
Birth Order	Re–coded into four categories: 1 for having first order birth, 2 for having second order birth, 3 for having third order birth, and 4 for having birth order four and above.
Ethnic Origin	Re-coded in four major ethnic groups: 4 for being Oromo, 3 for being Tigre, 2 for being Amhara and 1 for being other Ethiopian ethnic group categories as most of the other ethnic groups in this category are small in number.
Religion	Re-coded 1 for being Orthodox, 2 for being Muslim and 3 for being in other religious category (combining Protestant, Catholic, traditional and the other religious categories as most women in this category are small in number).
Maternal Education	Re-coded as no education, primary education and secondary or higher combining the secondary and higher education categories together.
Maternal Occupation	Re-coded as 1 for not working (combining it with domestic household work category), 2 for sales and services (combining sales, services and professional/technical/managerial categories), 3 for skilled manual, 4 for unskilled manual and agricultural laborers (combining unskilled manual categories, agricultural employee and self-employed categories).
Media Access	A composite index combining whether respondents read a newspaper or magazine, listen to radio and watch TV. Coded 1 for having no access to media,2 for having medium access to media (at least one) and 3 for having high access to media with access to more than one media at least once a week.
Wealth Index	The datasets contained wealth index that had been created using principal components analysis, coded 1 as poorest, 2 as poorer, 3 as middle, 4 as richer and 5 as richest.
Urban-rural residence	The variable place of residence in the dataset was retained without change and was re-coded 1 as rural and 2 urban.

### Data analysis

Analysis of socioeconomic inequalities was done using ADePT software (version 5). Living standard was measured by a composite variable “wealth index” available in the datasets as it stands out to be comparatively the most appropriate measure of socioeconomic status for national surveys compared to direct measures of living standard such as income, consumption or expenditure. Wealth index was constructed using information collected on durable asset ownership, access to utilities and infrastructure (e.g. ownership of television, sanitation facilities and source of water). Measurement of socioeconomic inequalities was done using concentration curves and concentration indices (CI). The curves plot the cumulative share of the health sector variable against the cumulative share of the living standard variable. In calculating the cumulative percentages, the socioeconomic variable has been ranked from lowest to highest. If the uptake of maternal health services is equally distributed, the curve will be running from the bottom left-hand corner to the top right-hand corner (a 45° line). This is known as the line of equality. By contrast, if the uptake of maternal health services is low among the poor, the concentration curve will lay below the line of equality [34, 35].

Concentration indices were used to describe the magnitude of inequalities. The index is defined as twice the area between the concentration curve and the line of equality (the 45° line). The index is 0 if there is no socioeconomic related inequality. The convention is that the index varies between −1 and 1 the interpretation of which is dependent on the selection of the outcome of interest. If the outcome is positive (e.g. uptake of maternal health services), that means the health variable is more concentrated among the non-poor and the concentration curve will lie below the line of equality. In contrast, a negative value means the health variable is more concentrated among the poor and the concentration curve will lie above the line of equality [34, 35].

For a discrete living standards variable, the index is defined as:

$$ C=\frac{2}{{\mathrm{n}\upmu}_h}{\displaystyle \sum_{i=1}^n}\ {\mathrm{h}}_i{\mathrm{R}}_i - 1 - \frac{1}{\mathrm{n}} $$

Where: h_*i*_ is the health sector variable, μ_*h*_ is its mean, and R_*i*_ = *i*/n is the fractional rank of individual *i* in the living standards distribution, with *i* = 1 for the poorest and *i* = n for the richest [30, 31]. The index summarizes information through the imposition of value judgments about the weight given to inequality at different points in the living standard distribution. The concentration index depends only on the relationship between health variable and the rank of the living standard variable (h_i_r_i_) and not on variation of living standard variable itself [34]. The value judgments implicit in the index are seen when the index is written as:

$$ C=1 - \frac{2}{n\ \upmu}{\displaystyle \sum_{i=1}^n}\ {\mathrm{h}}_i\ \left(1-{\mathrm{R}}_i\ \right) $$

The quantity h_*i*_/n μ is the i^th^ person share of uptake of maternal health services. This is then weighted in the summation by twice the complement of the person’s fractional rank, that is, 2 (1–R_*i*_). So the poorest person has the share of uptake weighted by a number close to two. The weights decline in a stepwise fashion, reaching a number close to 0 for the richest person. The extended concentration index is then 1 minus the sum of these weighted health shares.

1 $$ \mathrm{C}\;(v)=1-\frac{v}{n\upmu}{\displaystyle {\sum}_{i=1}^n\ {\mathrm{h}}_i{\left(1-{\mathrm{R}}_i\ \right)}^{\left(\  v-1\right)}} $$

Where: *v* is the inequality-aversion parameter (the weight attached to the i^th^ person’s health share), h_*i*_ /nμ,is now equal to *v* (1 – R_*i*_)^(*v* − 1)^, rather than by 2 (1–R_*i*_). When *v* = 1 everyone’s health is weighted equally. As $v$ is raised above 1, the weight attached to the health of a very poor person rises, and the weight attached to the health of a person above the 55^th^ percentile decreases [34, 35].

Achievement Index (AI) was used to reflect average level of the uptake of maternal health services and the inequality in health between the poor and the better off. The index is defined as a weighted average utilization of maternal health services in the sample with higher weights attached to the poor than to better-off. The index is given as:

$$ 1\;(v)=\frac{1}{n}{\displaystyle {\sum}_{i=1}^n{\mathrm{h}}_i\mathrm{v}\ {\left(1-{\mathrm{R}}_i\ \right)}^{\left(\  v-1\right)}} $$

This index can be shown to be equal to:

$$ \mathrm{I}\left(\mathrm{v}\right)=\mu \left[1 -\mathrm{C}\left(\mathrm{v}\right)\right] $$

When h is a measure of good health, high values of I (v) are considered good and C (v) > 0 (good health is higher among the non-poor). If the uptake of health services declines monotonically with living standard, the greater is the degree of inequality aversion, and the greater is the wedge between the mean (μ) and the value of the index I (v) [34, 35].

Indirect method of standardization was used to reflect differences across socioeconomic groups while controlling other determinants of the uptake of maternal health services. The standardizing variables are those correlated with the living standard measure and that of the maternal health service utilization outcomes from existing empirical literature. Such standardization provides a way to remove components of inequalities from socioeconomic related inequalities and describe the distribution of the maternal health service utilization outcomes by socioeconomic status conditional on other demographic, socio-cultural and environmental factors [34, 35].

$$ {\upgamma}_{\mathrm{i}}=\upalpha +{\displaystyle {\sum}_j\ \beta j\  Xij}+{\displaystyle {\sum}_k yk\kern0.24em  Zkj+{\varepsilon}_i} $$

Where: γ i is the health service utilization outcome for the *i* ^*th*^ individual; and α, β and γ are parameter vectors, x_j_ are confounding variables used to standardize, and z_k_ are non-confounding variables for which we do not want to standardize but do want to control for in order to estimate partial correlations with the confounding variables. α_,_ *βj and yk* parameter estimates of individual values of the confounding variables (x_ji_), and sample means of the non confounding variables (z _k_) are then used to obtain the predicted values of the health indicator γ_i_. Estimates of indirectly standardized health outcomes are then computed by the difference between actual and predicted outcomes plus the overall sample mean [34, 35].

Socioeconomic related inequalities were decomposed into the contributions of individual factors to wealth-related health inequality, in which each contribution is the product of the sensitivity of heath with respect to that factor and the degree of income-related inequality in that factor.

$$ {\mathrm{Y}}_{\mathrm{i}} = \alpha + {\displaystyle \sum } k\;{\beta}_{\mathrm{k}}{\mathrm{X}}_{\mathrm{k}\mathrm{i}} + {{\textsf{C}\hspace{-1.7ex}{=}}}_{\mathrm{i}} $$

Where: Y_i_ = 1 for the uptake of maternal health services, X_k_ a set of exogenous determinants of maternal health service utilization and β_k_ coefficient determinant X_k_, and €_i_ is random error term.

## Results

### Study population

Table 2 presents characteristics of the study population using three rounds of EDHS data (2000, 2005 and 2011). The study population is predominantly rural. This comprises well over 80% of the respondents across the surveys. About half of the respondents comprise those in the age group of 20–29 across the surveys. The general pattern of increase in the proportion of women with birth order reflects the high fertility regime in the country. Across the surveys, more than half of the respondents comprise those of birth order four and above. Among women of first order birth, the proportion declines to less than a fifth of the respondents.

**Table 2:** Distribution of Women by Demographic and Socioeconomic Characteristics by Survey Year

Women’s Characteristics	Year 2000 (*N* = 7917)		Year 2005 (*N* = 7273)		Year 2011 (*N* = 7836)	
Age group	Frequency	Percent	Frequency	Percent	Frequency	Percent
15–19	468	5.9	430	5.9	393	5.0
20–29	3703	46.8	3416	47.0	3936	50.2
30–39	2710	34.2	2561	35.2	2721	34.7
40–49	1036	13.1	866	11.9	787	10.0
Birth order						
1st order birth	1313	16.6	1163	16.0	1346	17.2
2nd order birth	1274	16.1	1082	14.9	1337	17.1
3rd order birth	1087	13.7	1000	13.8	1109	14.2
Birth order 4+	4243	53.6	4028	55.4	4044	51.6
Education						
No education	6524	82.4	5723	78.7	5246	66.9
Primary	993	12.5	1189	16.3	2236	28.5
Secondary or higher	401	5.1	361	5.0	355	4.5
Occupation						
Not working	2756	34.8	5014	68.9	3486	44.5
Sales and Services	901	11.4	624	8.6	1464	18.7
Skilled manual	664	8.4	113	1.6	558	7.1
Unskilled manual and Agri workers	3587	45.3	1509	20.7	2252	28.7
Ethnic origin						
Other Ethnic Ethiopians	2108	26.6	2173	29.9	2305	29.4
Amhara	2529	31.9	2117	29.1	2232	28.5
Oromo	2734	34.5	2499	34.4	2740	35.0
Tigray	542	6.8	467	6.4	517	6.6
Religion						
Orthodox	4012	50.7	3233	44.5	3266	41.7
Muslim	2330	29.4	2382	32.7	2562	32.7
Other religions	1576	19.9	1658	22.8	1916	24.4
Media Access						
No access	5775	72.9	4560	62.7	3154	40.2
Moderate access	2064	26.1	2573	35.4	4112	52.5
High access	69	.9	111	1.5	554	7.1
Urban-rural residence						
Urban	875	11.1	621	8.5	1160	14.8
Rural	7042	88.9	6652	91.5	6676	85.2

Although the proportion of women with no education declines from 75% in the year 2000 to 51% in the year 2011, the education profile of the study population shows the vast majority of the respondents have no formal education. Nearly, three in ten (in the year 2000) and more than four in ten of the women comprise the not working category in the year 2005 and 2011. Followed by other ethnic Ethiopians and the Amharas, ethnic Oromos comprise the largest number of the respondents. Women in this ethnic category comprise 35, 34 and 35% of the respondents in the year 2000, 2005 and 2011. Although Muslim women comprise a significant proportion of the respondents (see the table below), the study sample is predominantly Orthodox Christian. Women in this category comprise 51, 46 and 41% of the respondents in the year 2000, 2005 and 2011.

Table 3 below presents the levels, patterns and trends of maternal health service utilization by wealth in Ethiopia across the three surveys. As shown in the Table, the uptake of ANC and delivery care is quite low in the study area. Among those who had a live birth in the 5 years preceding the surveys, slightly over a quarter of them received a minimum of one ANC from skilled provider (doctor, nurse or midwife) in the year 2000 and 2005. By comparison, almost a third of the women received a minimum of one ANC in the year 2011. This corresponds to 31% increase in the uptake between the year 2000 and 2011. Coverage of key components of ANC and delivery care also shows a similar trend. The uptake of a minimum of four ANC increased by 73% from the level of one in ten women in the year 2000 to one in every five women in the year 2011.

**Table 3:** Uptake of Maternal Health Services by Wealth Status (Year 2000, 2005 & 2011)

Wealth Status	Minimum of one ANC	Four or more ANCs	First trimester initiation of ANC	Two or more doses of TT injection	Delivery in health facilities	Skilled delivery assistant
Year 2000						
Poorest	0.180	0.077	0.237	0.152	0.041	0.046
Poorer	0.200	0.075	0.214	0.159	0.038	0.042
Middle	0.230	0.084	0.210	0.151	0.032	0.036
Richer	0.303	0.086	0.195	0.181	0.033	0.034
Richest	0.524	0.296	0.260	0.318	0.173	0.194
Ethiopia	0.267	0.110	0.227	0.182	0.055	0.061
Year 2005						
Poorest	0.163	0.078	0.209	0.217	0.052	0.048
Poorer	0.230	0.100	0.181	0.249	0.048	0.046
Middle	0.278	0.104	0.199	0.289	0.034	0.035
Richer	0.345	0.124	0.187	0.356	0.057	0.061
Richest	0.502	0.332	0.314	0.428	0.247	0.256
Ethiopia	0.276	0.125	0.223	0.290	0.068	0.069
Year 2011						
Poorest	0.228	0.134	0.244	0.275	0.081	0.073
Poorer	0.279	0.149	0.208	0.343	0.071	0.066
Middle	0.286	0.172	0.231	0.341	0.066	0.061
Richer	0.444	0.230	0.251	0.405	0.113	0.112
Richest	0.653	0.409	0.354	0.490	0.436	0.443
Ethiopia	0.343	0.196	0.260	0.355	0.122	0.119

The uptake of two or more doses of TT injection also increased by 94% from 18 to 35% for the same period. Although to a lesser extent, initiation of the first ANC in the first trimester of pregnancy also increased by 13% from the level of 23% the year 2000 to 26% in the year 2011. In contrast, births delivered in health facilities more than doubled increasing from nearly 5 to 12% between the year 2000 and 2011. Furthermore, births delivered with the assistance of skilled health personnel also doubled from its level of 6 to 12%. Also evident, in almost all the cases, most of the increase is noted between the year 2005 and 2011 (see Table 3. below).

The uptake of maternal health services by wealth as shown in the Table 3 also reveals a consistent direct concentration pattern with wealth status across the surveys. For example, the uptake of a minimum of one ANC and adequate ANC (3+ ANC) were more frequent among the richest segment of society. Initiation of the first ANC in the first trimester of pregnancy and the uptake of two or more doses of TT injection were also varied by wealth status favoring the non-poor. Among the outcomes considered, a rather stark contrast was noted in the uptake of delivery care by wealth quintile which also increased with wealth status.

Moreover, across the survey years, the average increase in the uptake of these services is greater among women in the richest wealth quintile. Likewise, initiation of the first ANC in the first trimester of pregnancy amongst the poorest wealth quintile increased marginally, whereas the increase for women in the richest wealth quintile was greater. Furthermore, the absolute increase in the uptake of two or more doses of TT injection was greater for women living in the wealthiest households. Similarly, across the survey years, the increase in uptake of delivery care was negligible for the poorest households compared to the substantial increase in uptake of such services by women in the richest wealth quintile particularly after 2005.

### Inequalities in uptake of maternal health services in Ethiopia

**Fig. 1:**
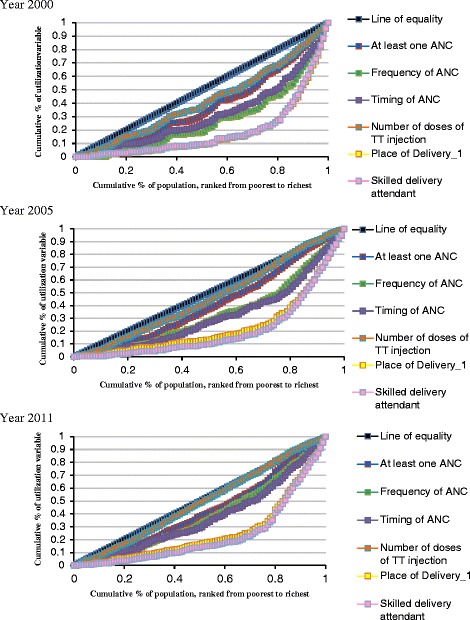
Concentration Curves for Uptake of Maternal Health Service (Year 2000, 2005 and 2011)

Figure 1 shows the concentration curves plotting the cumulative share of selected maternal health service utilization outcomes against the proportional cumulative economical levels across the three survey years. All curves lie below the line of equality, which confirms that the uptake of maternal health services is inversely proportional to wealth status. Further shown in the graph is that inequality was more prevalent in the uptake of delivery care than the other maternal health service utilization outcomes. The curves for indicators associated with delivery care were further away from the line of equality across the three surveys. In contrast, the concentration curve for TT injection almost overlaps with the line of equality such as in the year 2011 the wealth-related difference in uptake of two or more doses of TT injection was negligible.

Results of the inequality statistics estimated by concentration indices (CI) are presented in Table 4. The positive CI values of 0.308, 0.323 and 0.310 for uptake of a minimum of one ANC in the year 2000, 2005 and 2011 respectively indicate skewness towards the non-poor population. Moreover, there appears a marginal increase in CI in the year 2011 compared to the year 2000 which is suggestive of the persistent inequity with no sign of decline over the study period. Interestingly, inequality in the uptake of four or more ANC declined from 0.409 in the year 2000 to 0.353 in 2011. A decline in inequality was also observed in terms of uptake of two or more doses of TT injection over the study period. The CI for TT injection declined from 0.270 to 0.222.

**Table 4:** Inequalities in Uptake of Maternal Health Services in Ethiopia (Year 2000, 2005 & 2011)

Year	Minimum of one ANC	Four or more ANCs	First trimester initiation of ANC	Two or more doses of TT injection	Delivery in health facilities	Skilled delivery assistant
Year 2000						
Standard concentration index	0.308	0.409	0.253	0.270	0.492	0.478
CI with inequality-aversion parameter = 3	0.409	0.479	0.255	0.346	0.518	0.508
CI with inequality-aversion parameter = 4	0.478	0.533	0.258	0.409	0.552	0.543
Standard achievement index	0.184	0.065	0.170	0.132	0.027	0.031
AI with inequality-aversion parameter = 3	0.157	0.057	0.169	0.119	0.026	0.030
AI with inequality-aversion parameter = 4	0.139	0.051	0.168	0.107	0.024	0.027
Year 2005						
Standard concentration index	0.323	0.404	0.291	0.257	0.483	0.515
CI with inequality-aversion parameter = 3	0.444	0.489	0.315	0.359	0.528	0.569
CI with inequality-aversion parameter = 4	0.523	0.551	0.332	0.433	0.566	0.608
Standard achievement index	0.187	0.074	0.158	0.216	0.035	0.033
AI with inequality-aversion parameter = 3	0.153	0.063	0.153	0.186	0.032	0.029
AI with inequality-aversion parameter = 4	0.131	0.056	0.149	0.164	0.029	0.027
Year 2011						
Standard concentration index	0.310	0.353	0.267	0.222	0.501	0.527
CI with inequality-aversion parameter = 3	0.424	0.453	0.312	0.319	0.575	0.606
CI with inequality-aversion parameter = 4	0.499	0.522	0.345	0.396	0.621	0.652
Standard achievement index	0.236	0.127	0.191	0.276	0.061	0.056
AI with inequality-aversion parameter = 3	0.197	0.107	0.179	0.241	0.052	0.046
AI with inequality-aversion parameter = 4	0.171	0.093	0.170	0.214	0.046	0.041

In contrast, an increase in inequality was observed for initiation of the first ANC in the first trimester of pregnancy from 0.253 to 0.267. Similarly, inequalities in the uptake of health facilities for delivery and assistance from skilled health personnel also went up. CI for uptake of health facilities for delivery grew from 0.492 to 0.501 and the CI for delivery with skilled assistance increased from 0.478 to 0.527. This means that inequalities in uptake of these services except as in cases of the uptake of four or more ANC and two or more doses of TT injection has rather widened in favor of the better-offs over the study period. Furthermore, CI values across the surveys also grew more positive with an increase in inequality aversion parameters which further confirms unequal uptake of these service favoring the non-poor even when higher weights are attached to the poor than to the better-offs.

On the other hand, there is a general upward trend in the weighted average of utilization of maternal health services over the 11-year period. The standard achievement index for the selected outcomes increased by the year 2011 from the level observed in the year 2000. This suggests the increase in the average level of maternal health service utilization over the study period. However, values of achievement index (AI) decreased with an increase in inequality aversion parameter. This means even if average level of utilization of maternal health services increased over the study period, with greater weight assigned to the poorest wealth quintile, greater increase in the level of uptake of such services went to the richest segment of society compared to the poorer ones.

### Decomposition analysis

Decomposition analysis was employed to identify dominant factors contributing for the observed inequalities in the uptake of maternal health services. Utilization of maternal health services might be lower, for example, among the poor because in part the poor are on average less educated, have higher fertility, engage in poverty induced jobs, reside in rural areas, or might belong to a certain ethnic and/or religious groups. For the decomposition analysis, age, birth order, maternal education, occupation, ethnic origin, religious affiliation and media access were used as need/standardizing variables.^1^

Table 5 below  presents concentration indices for standardizing and control variables for the survey year 2000, 2005 and 2011. Accordingly, for the year 2000, higher birth order women and those in the not working categories were seem to have more concentrated among the poor and these variables have negative concentration indices, but changed in later period. On the other hand, women who are more educated and those with access to media seem to live in richer households and these variables have positive concentration indices. The concentration index for wealth status is the Gini coefficient, as observations are ranked according to wealth quintile. The CI estimates for the years 2000 are same in all the maternal healthcare indicators except in initiation of the first ANC in the first trimester of pregnancy. The same patterns continued to the year 2005 and 2011 whereas, women of more educated, working mothers and those with access to media are mostly living in richer households and therefore these variables have positive concentration indices, favoring the rich.

**Table 5:** Concentration Indices of Covariates (Year 2000, 2005 & 2011)

Standardizing (need) variables	A minimum of one ANC	Four or more ANC	First trimester initiation of ANC	Two or more doses of TT injection	Delivery in health facilities	Skilled delivery assistant
	coef	coef	coef	coef	coef	coef
Year 2000						
Age in group	0.007	0.008	0.019	0.008	0.008	0.007
Birth order	−0.002	−0.001	0.012	−0.001	−0.001	−0.002
Educational level	0.571	0.571	0.520	0.571	0.571	0.571
Maternal occupation	−0.018	−0.018	−0.037	−0.018	−0.018	−0.018
Ethnic Origin	0.052	0.052	0.066	0.052	0.052	0.052
Religion	0.032	0.032	0.013	0.032	0.032	0.032
Media access	0.120	0.120	0.173	0.120	0.120	0.120
Control variable						
Wealth Status	0.267	0.267	0.214	0.267	0.267	0.267
Year 2005						
Age in group	0.026	0.026	0.027	0.026	0.026	0.026
Birth order	0.020	0.020	0.014	0.020	0.020	0.020
Educational level	0.571	0.572	0.536	0.572	0.572	0.571
Occupation	0.132	0.132	0.055	0.132	0.132	0.132
Ethnic Origin	0.024	0.024	0.023	0.024	0.024	0.024
Religion	0.033	0.033	0.019	0.033	0.033	0.032
Media access	0.408	0.407	0.365	0.407	0.407	0.409
Control variable						
Wealth Status	0.267	0.267	0.209	0.267	0.267	0.267
Year 2011						
Age in group	0.024	0.024	0.017	0.024	0.024	0.024
Birth order	0.013	0.013	−0.004	0.013	0.013	0.013
Educational level	0.449	0.448	0.415	0.448	0.448	0.448
Occupation	0.025	0.025	−0.041	0.025	0.025	0.025
Ethnic Origin	0.051	0.051	0.042	0.051	0.051	0.051
Religion	0.029	0.029	0.016	0.029	0.029	0.029
Media access	0.112	0.112	0.116	0.112	0.112	0.112
Control variable						
Wealth Status	0.272	0.272	0.231	0.272	0.272	0.272

## Discussion

The CI approach complemented with concentration curves has allowed the examination of trends in socio-economic inequities in the uptake of maternal health services in Ethiopia over a period of 11 years (2000–2011). The results further show the distribution of uptake of maternal health services is inequitable to the disadvantage of the poor. The concentration indices of all the outcomes of interest are positive revealing higher utilization of maternal health services by richer women and the poor–rich gap in delivery care are much larger than those in the other forms of care. This is consistent with other studies [31, 36–38].

The analysis further showed trends in wealth related inequities in the uptake of maternal health services varied over time. For example, despite encouraging improvement in the uptake of maternal health services over the study period and that progress has been made in reducing inequalities in the uptake of four or more ANC and two or more doses of TT injection over the 11 year period, inequalities in the uptake of health facilities for delivery and skilled assistance during delivery has widened over the same period. In equity terms, the performance achieved in improving uptake of four or more ANC and receipt of two or more doses of TT injection could be the outcome of the health extension program launched in the year 2003. Thus, it can be argued that the program has promoted public awareness on the need to attend maternal health services during pregnancy among the rural populace and those in the lower sections of society [39, 40].

The observed widening inequalities in the uptake of delivery care favoring the rich over the study period, however, mean the implemented health programs have not adequately addressed the issue of equity. Possible explanation for this could be the presence of informal fees for delivery-related services on supplies such as laboratory services, gloves, glucose and drugs that could have made the uptake health facilities for delivery and skilled assistance during delivery difficult for the poorest segments of society due to the resulting out of pocket expenditure in seeking such services [41]. For example, a study of user fees and maternity services in Ethiopia found that among facilities that provided delivery care, 68% charged fees for drugs and supplies in cash or in kind and the average cost of normal and caesarean delivery was reported US$ 7.00 and US$ 51.80 respectively [42].

The results of the decomposition analysis across the survey years further show wealth status seems to be an important positive contributor to the concentration indices of the six health service utilization outcomes. This means inequality in wealth makes health service utilization more predominant among richer individuals. Furthermore, inequality in education and media access makes the concentration index pro-rich. This means access to media and even more so education makes maternal health service utilization more frequent among richer individuals.

Other studies also show similar results [36–38] the explanation of which being income and assets are a key component of household resources that enable individuals to access health services. The human capital of knowledge and education also tend to be positively associated with household wealth. As evident in the relative contribution of the standardizing or need variables to wealth related inequalities, the decomposition analysis showed inequalities in mother’s education and household access to media together accounted for a large share of wealth related inequality in the uptake of maternal health services in Ethiopia. However, in respect to contributions, household wealth was the most important. Thus, it can be argued that the inequitable distribution of uptake of maternal health services to the disadvantages of the poor is not just a matter of the better-offs using higher incomes to access care but also due to the contribution to inequality in health service utilization by education and access to media favoring the non-poor.

## Conclusion

Uptake of antenatal care and delivery care is extremely low in Ethiopia. This highlights the need to improve coverage of these services in the country. The evidence that utilization of maternal health services is inequitable, to the disadvantage of the poor, also suggests that improving the uptake of maternal health services in Ethiopia goes beyond improving coverage of health service and requires better planning to ensuring equitable provision of maternal health services in the country. Furthermore, the most inequitable interventions should receive attention to ensure that all social groups are reached. Finally, as Ethiopia moves forward with the sustainable development agenda, socioeconomic inequalities in uptake of maternal health services should be continuously monitored.

### Study limitation

Certain limitations are worth mentioning. First, the cross-sectional nature of the data does not allow drawing causal inferences. Second, DHS data are associated with recall bias given that data was collected retrospectively on events that took place 5 years before the survey.

## Footnotes

1 Type of place of residence was not used as a standardizing variable in the analysis. As Barros AJD, Victoria CG (2013) indicated among the limitations of using asset indices as socio-economic position indicators is families in the wealthiest quintile in most low- and middle-income countries are mainly from urban areas that results in highly unbalanced groups and wealth inequalities are closely associated with urban/ rural disparities. In the Ethiopian case, the distribution of wealth quintile varies sharply by type of place of residence. For example, while 88% of the urban population in Ethiopia is in the highest wealth quintile, only 5% of the rural population is in the highest wealth quintile in the year 2011. This comprises an insignificant proportion given that the country is one of the least urbanized countries with only 16% of the population living in urban areas (CSA, 2011). Murray, C.J.L., Gakidou, E.E,.&Frenk, J. (1999) further noted when the geographical areas used to categorize the population are small and represent relatively homogeneous groups, categorization of the population by these measures provide results close to revealing the extent of individual level variation in the population.
